# IgG4-related lung disease with recurrent pulmonary lesions during steroid therapy and difficulty in differentiating from malignancy: a case report

**DOI:** 10.1186/s13019-022-01805-x

**Published:** 2022-04-01

**Authors:** Tomohito Okubo, Nariyasu Nakashima, Yoshimasa Tokunaga, Nobuyuki Kita, Hiroyuki Nakamura, Ryou Ishikawa, Setsuo Okada, Tetsuhiko Go, Hiroyasu Yokomise

**Affiliations:** 1 Department of General Thoracic Surgery, Sakaide City Hospital, 3-1-2, Kotobuki-chou, , Sakaide-shi, Kagawa-ken 762-8550 Japan; 2 Department of General Thoracic Surgery, KKR Takamatsu Hospital, 4-18, Tenjinmae, Takamatsu-shi, Kagawa-ken 760-0018 Japan; 3 Department of Respiratory Medicine, Sakaide City Hospital, 3-1-2, Kotobuki-chou, , Sakaide-shi, Kagawa-ken 762-8550 Japan; 4 Department of Diagnostic Pathology, Kagawa University Hospital, 1750-1, Ikenobe, Miki-chou, Kita-gun, Kagawa-ken 761-0793 Japan; 5 Department of Surgery, Sakaide City Hospital, 3-1-2, Kotobuki-chou , Sakaide-shi, Kagawa-ken 762-8550 Japan; 6 Department of General Thoracic Surgery, Kagawa University Hospital, 1750-1, Ikenobe, Miki-chou, Kita-gun, Kagawa-ken 761-0793 Japan

**Keywords:** IgG4-related lung disease, Lung mass, Multiple lung nodules, Thoracoscopic lung biopsy, Thoracoscopic partial lung resection, Case report

## Abstract

**Background:**

Immunoglobulin G4-related disease (IgG4-RD) is characterized by the formation of inflammatory lesions with fibrosis and infiltration of IgG4-positive plasma cells and lymphocytes in various organs of the body. Since the first report of IgG4-related autoimmune pancreatitis, IgG4-RD affecting various organs has been reported; however, only a few reports of IgG4-related lung disease (IgG4-RLD) exist. In this report, we describe a case of IgG4-RLD that was difficult to differentiate from malignancy, and the usefulness of the surgical approach in determining the appropriate diagnosis and treatment plan.

**Case presentation:**

A 61-year-old man was referred to our hospital after a chest radiograph revealed an abnormal chest shadow. At the time of his first visit, he had a slight fever and dyspnea on exertion. Chest computed tomography (CT) revealed a middle lobe hilar mass with irregular margins and swelling of the right hilar and mediastinal lymph nodes. These findings were not present on CT 1.5 years ago. ^18^F-fluorodeoxyglucose-positron emission tomography revealed a mass lesion with a maximum diameter of 5.5 cm, maximum standardized uptake value (SUVmax) of 11.0, and areas with high SUV in the hilar and mediastinal lymph nodes. We suspected lung cancer or malignant lymphoma and performed a thoracoscopic lung biopsy to confirm the diagnosis. Histopathological examination revealed no malignant findings, and IgG4-RLD was diagnosed. One month after treatment with prednisolone (PSL), the tumor had shrunk, but a CT scan during the third month of PSL treatment revealed multiple nodular shadows in both lungs. Considering the possibility of malignant complications and multiple lung metastases, we performed thoracoscopic partial lung resection of the new left lung nodules to determine the treatment strategy. Histopathological examination revealed no malignant findings in any of the lesions, and the patient was diagnosed with IgG4-RLD refractory to PSL monotherapy.

**Conclusions:**

IgG4-RLD refractory to PSL monotherapy showed changes from a solitary large mass (pseudotumor) to multiple nodules on chest CT. It was difficult to distinguish malignancy from IgG4-RLD based on imaging tests and blood samples alone, and the surgical approach was useful in determining the appropriate diagnosis and treatment plan.

## Background

Since autoimmune pancreatitis with high serum immunoglobulin (Ig) G4 concentrations was reported in 2001, the new disease concept of IgG4-related disease (IgG4-RD) has been widely recognized [1]. IgG4-RD is characterized by the development of inflammatory lesions in various organs of the body; however, there are only a few reports on the clinical course of pulmonary lesions. In this report, we describe a case of IgG4-related lung disease (RLD) with recurrent pulmonary lesions during steroid therapy that was difficult to differentiate from malignancy, and the surgical approach was useful in determining the appropriate diagnosis and treatment plan. This case report is in line with the SCARE Criteria.

## Case presentation

**Fig. 1:**
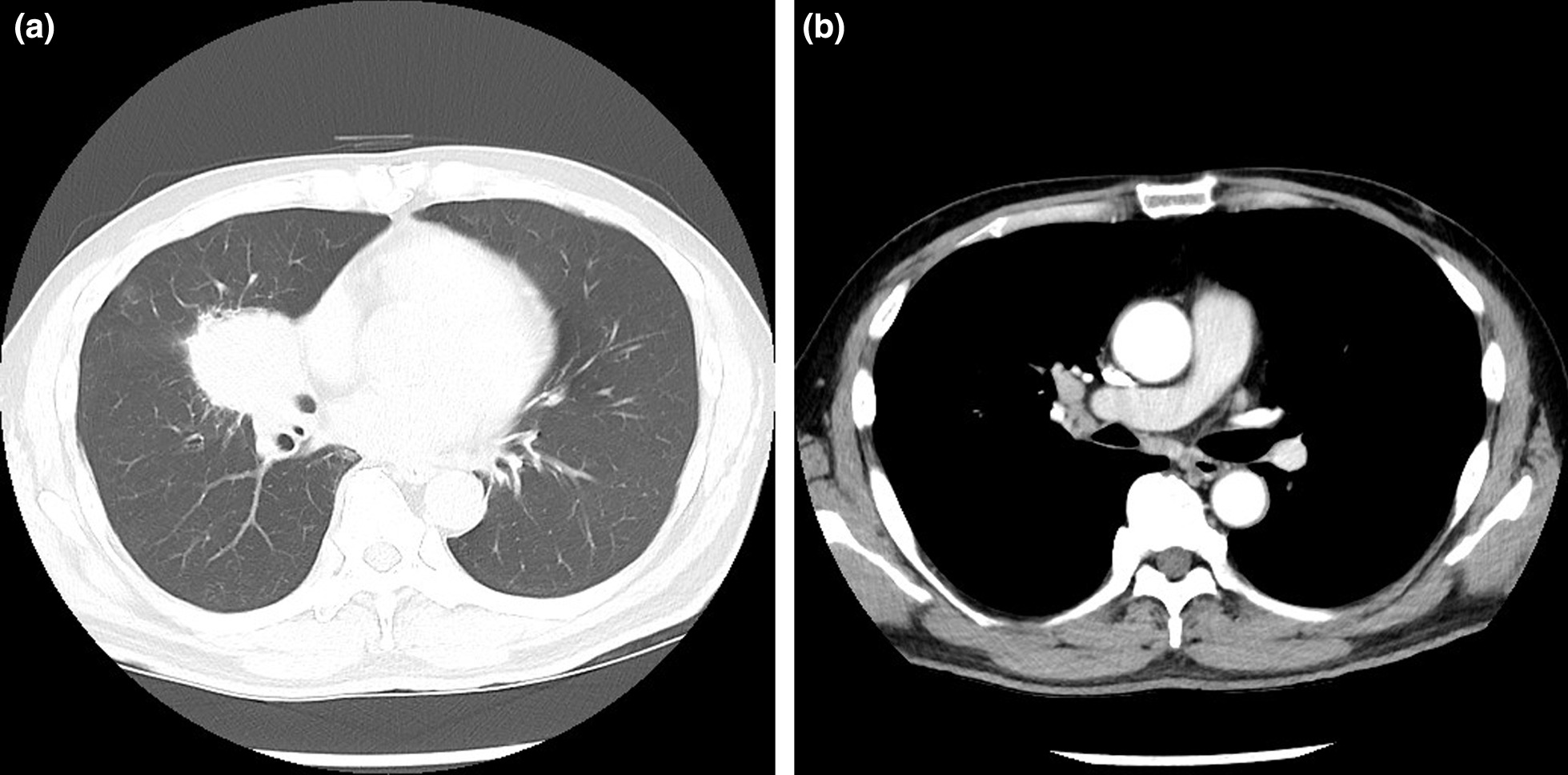
Chest CT. **a**, **b** Chest CT revealed a middle lobe hilar mass with irregular margins and swelling of the right hilar and mediastinal lymph nodes

**Fig. 2:**
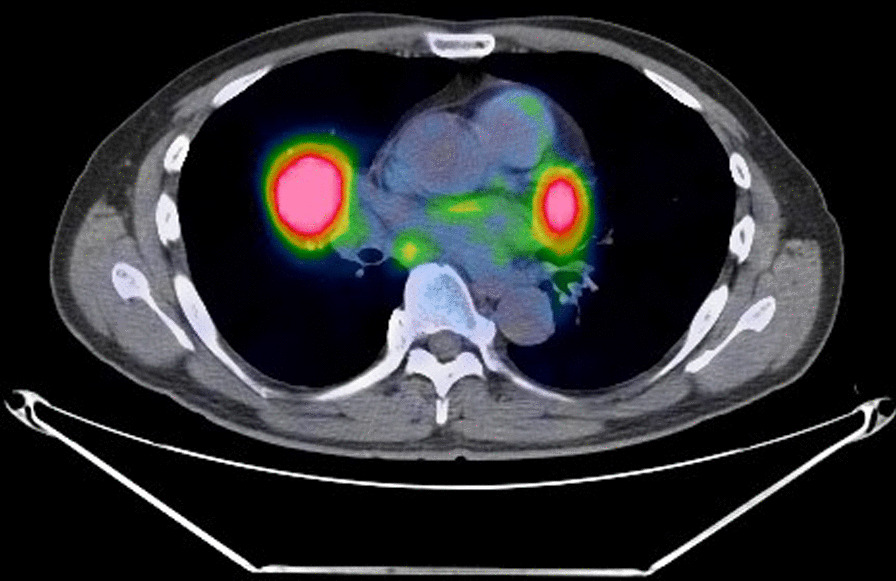
FDG-PET/CT. FDG-PET/CT revealed a mass lesion with a maximum diameter of 5.5 cm, SUVmax of 11.0, and high SUV areas in the hilar and mediastinal lymph nodes

A 61-year-old man undergoing treatment for bronchial asthma was referred to our hospital because of an abnormal mass shadow on chest radiography. At the time of his first visit, he had a fever of approximately 37 °C, accompanied by persistent fatigue and mild dyspnea on exertion. He had a history of bronchial asthma, hypertension, and dyslipidemia, and was under medical treatment. He had no history of allergies and had smoked 20 cigarettes a day for 7 years between the ages of 18 and 25 years. He had worked as a pilot, but after retiring, he worked as a tree trimmer. Blood tests showed a mild increase in C-reactive protein levels to 3.5 mg/dL, but no other abnormalities in blood count or biochemical findings were observed. No elevation of tumor markers related to lung cancer was observed, but interleukin (IL)-2R was elevated to 536 U/mL (normal value 124–466 U/mL). Electrocardiography and echocardiography revealed no abnormalities. Chest computed tomography (CT) showed a middle lobe hilar mass with irregular margins and swelling of the right hilar and mediastinal lymph nodes (Fig. 1). ^18^F-fluorodeoxyglucose-positron emission tomography (FDG-PET)/CT revealed a mass lesion with a maximum diameter of 5.5 cm and maximum standardized uptake value (SUVmax) of 11.0, and high SUV areas in the hilar and mediastinal lymph nodes (Fig. 2). Therefore, we included middle lobe lung cancer with mediastinal lymph node metastasis (T3N2M0 Stage IIIB) and malignant lymphoma in the differential diagnosis. Transbronchial lung biopsy of the mass showed no malignant findings. Based on the above, we decided to perform a thoracoscopic lung biopsy; if intraoperative histology revealed malignant findings, a radical middle lobectomy plus lymph node dissection was planned.

### Surgical findings

The surgery was performed under general anesthesia, including differential lung ventilation and left lower lateral recumbency. A 1.5-cm camera port was made on the mid-axillary line of the seventh intercostal space, and the thoracic cavity was observed; however, it was difficult to evaluate the pleural effusion due to extensive pleural adhesions. The finding of firm adhesions with loss of normal laminar structure, which is atypical for cancer invasion, mainly in the oblique fissure near the tumor was striking. In addition, the normal layered structure of the vascular sheath of the pulmonary artery was also not preserved, making dissection difficult. After thoracoscopic dissection of the pleural adhesions, a total of five tumor sites and an enlarged lymph node (LN#10) were biopsied; however, intraoperative rapid pathological examination revealed no malignant findings. Dissection of the interlobar pulmonary artery was impossible due to severe interlobar adhesions, and a right total pneumonectomy was necessary to completely resect the primary lesion. Since it would be over-invasive to perform a total right pneumonectomy in a patient with an undetermined diagnosis, we decided to terminate the surgery and determine the treatment plan based on the results of the histopathological examination. The duration of the surgery was 256 min, and total blood loss was 80 mL. No postoperative complications were observed.

### Histopathological findings

**Fig. 3:**
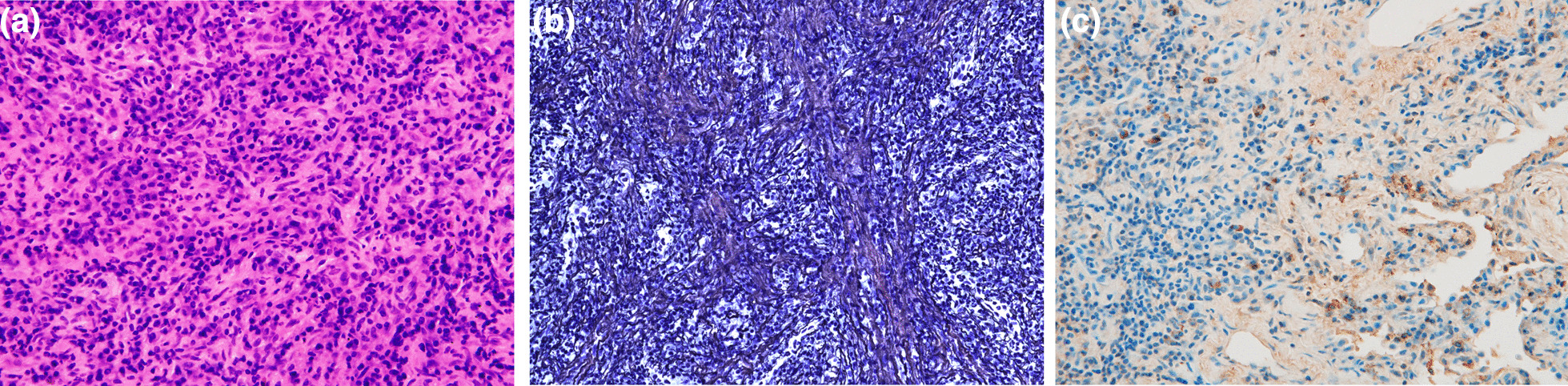
Microscopic examination. **a** HE staining showed an inflammatory cell infiltrate consisting of plasma cells, lymphocytes, and neutrophils. **b** Storiform fibrosis was observed using the silver impregnation method. **c** Immunohistochemical staining showed that there were approximately 40 IgG4-positive cells per HPF, and the IgG4/IgG ratio was approximately 20%

Hematoxylin–eosin (HE) staining showed an inflammatory cell infiltrate consisting of plasma cells, lymphocytes, and neutrophils. Storiform fibrosis was observed using silver impregnation. Immunohistochemical staining showed that there were approximately 40 IgG4-positive cells per high-power field (HPF), and the IgG4/IgG ratio was approximately 20% (Fig. 3).

**Fig. 4:**
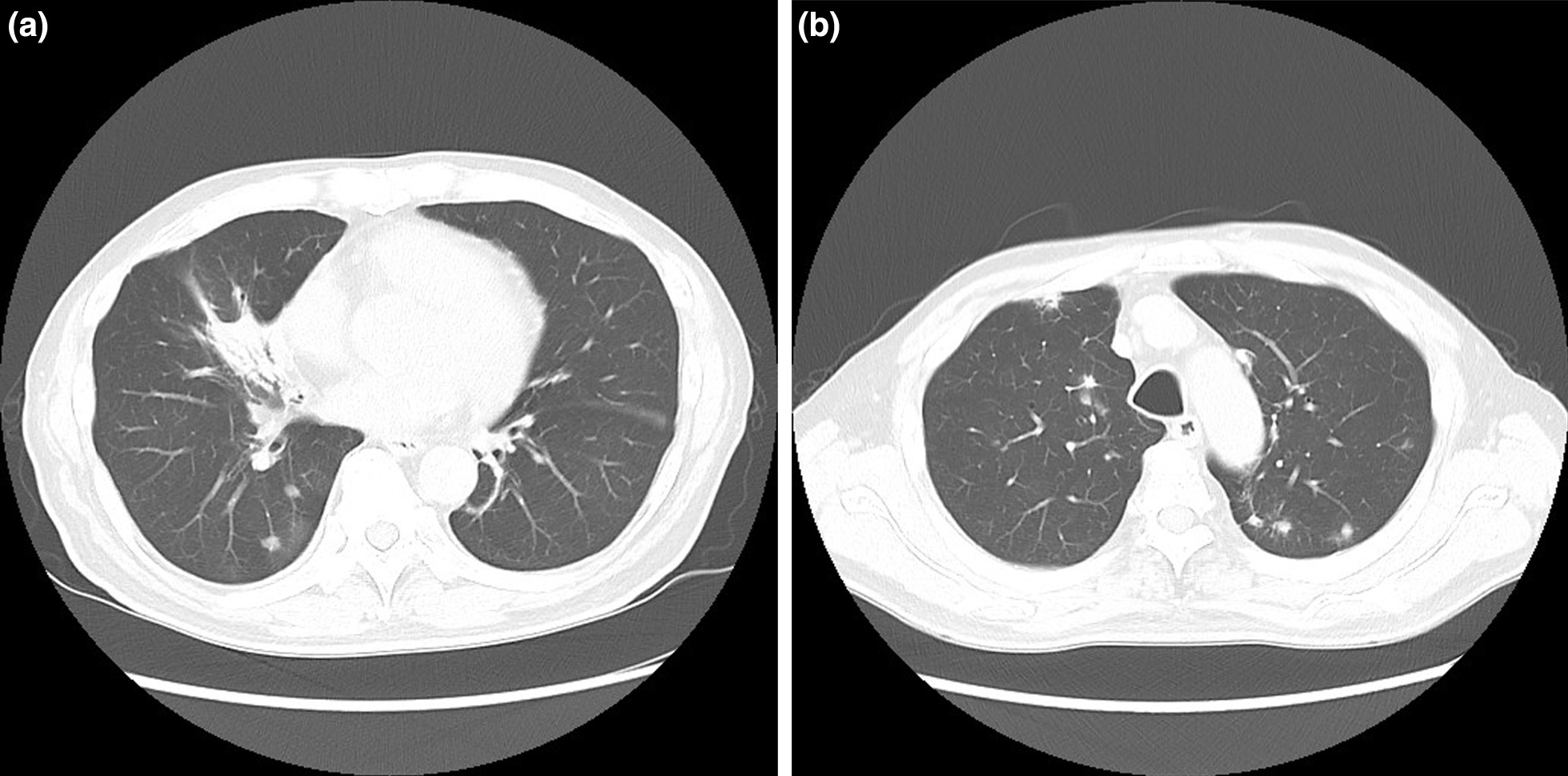
Chest CT view at the third month of PSL treatment (30 mg/day). **a**, **b** Chest CT showing a shrinking middle lobe hilar mass and multiple nodular shadows in both lungs

Based on the above findings, the patient was diagnosed with IgG4-RD occurring only in the lungs, and treatment with intravenous prednisolone (PSL) 60 mg/day was initiated. After 1 month of treatment with PSL, the mass had shrunk, and improvement in fever and cough symptoms was observed. The steroid dose was gradually reduced and changed to 30 mg/day orally in the third month after the start of therapy. During this period, there were no PSL administration errors, and oral administration was accurate. However, a CT scan during the third month of PSL treatment (30 mg/day) showed multiple nodular shadows in both lungs (Fig. 4). No physical symptoms were observed. Considering the possibility of malignant complications and multiple lung metastases, we performed thoracoscopic partial lung resection of the newly appearing left lung nodules to determine the treatment strategy.

### Surgical findings

The surgery was performed under general anesthesia, including differential lung ventilation and right lower lateral recumbency. We performed 3-port video-assisted thoracic surgery (VATS). Extensive pleural adhesions were observed in the thoracic cavity, but the adhesions were sufficiently detachable compared to right-sided surgery. After detachment of the adhesions, the surgery was completed with partial lung resection of the lower lobe. The surgery duration was 126 min, and the total blood loss was 10 mL. No postoperative complications were observed.

### Histopathological findings

**Fig. 5:**
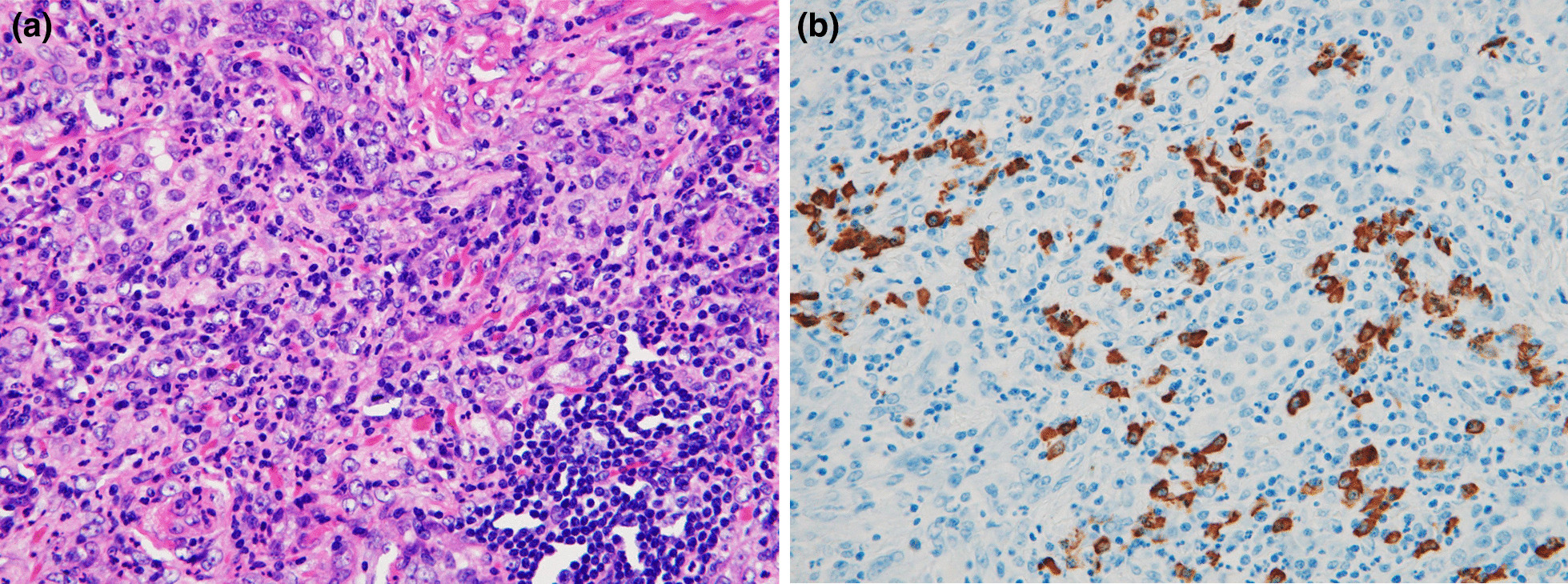
Microscopic examination.** a** HE staining showing a dense inflammatory cell infiltrate consisting of plasma cells, lymphocytes, and neutrophils. **b** Immunohistochemical staining showing approximately 40 IgG4-positive cells per HPF, and an IgG4/IgG ratio of approximately 70%

HE staining revealed a dense inflammatory cell infiltrate consisting of plasma cells, lymphocytes, and neutrophils. Immunohistochemical staining showed that there were approximately 40 IgG4-positive cells per HPF, and the IgG4/IgG ratio was approximately 70% (Fig. 5). There was no evidence of malignancy.

Thus, we diagnosed IgG4-RLD refractory to PSL monotherapy.

## Discussion

IgG4-RD is a relatively new disease concept that was historically proposed based on observations of autoimmune pancreatitis and Mikulicz’s disease with high IgG4 levels in the serum [1, 2]. This disease is characterized by the formation of inflammatory lesions with fibrosis and infiltration of IgG4-positive plasma cells and lymphocytes in various organs (e.g., pancreas, biliary tract, lacrimal gland, salivary gland, kidney, and retroperitoneum). Although the frequency of lung or pleural lesions in IgG4-RD has been reported to be 14%, there are still only a few reports of IgG4-RLD [3]. The clinical symptoms may be nonspecific, such as cough, respiratory distress, fever, and chest pain, but may also be asymptomatic. The ACR/EULAR IgG4-RD classification criteria were developed and proposed in 2019 as diagnostic criteria [4]. According to the IgG4-RD classification criteria proposed by Wallace et al., the diagnostic criteria are as follows: pathological tissue score, immunohistochemistry (IHC) score, serum IgG4, and findings in each organ. In this patient, the pathological tissue score was 13 points, the IHC score was 7 points, the serum IgG4 was 0 points, and the organs were 4 points, for a total of 24 points according to the test results when VATS lung biopsy was performed. At the time of VATS partial lung resection, the test results were 4 points for the pathological tissue score, 14 points for the *IHC* score, 0 points for serum IgG4, and 4 points for organs, for a total of 22 points. Since this case met the diagnostic criteria of > 20 points, the patient was diagnosed with IgG4-RD. Epidemiologically, it is more common among middle-aged and older men and varies from systemic to single-organ diseases. According to previous reports, imaging findings such as consolidation, ground-glass opacity, nodular shadows, and septal thickening are often seen in the peripheral lung fields, and abnormalities are often seen just below the pleura on chest CT. Fibrosis has been observed but is less common. The presence of fibrosis may be accompanied by honeycombing and traction bronchiectasis [5].

In our case, FDG-PET/CT scans showed a mass lesion measuring 5.5 cm in size in the middle lobe with an SUVmax of 11.0, and high SUV areas in the hilar and mediastinal lymph nodes. IL-2R levels were elevated, and it was difficult to differentiate malignant tumors from benign lung disease based on preoperative examination. Thoracoscopic lung biopsy was performed, which led to the diagnosis of IgG4-RLD with minimal invasion. Intraoperative findings were characterized by firm adhesions with loss of normal laminae, which is atypical for cancer invasion, suggesting a severe inflammatory reaction around the tumor. In general, glucocorticoids are effective in the treatment of IgG4-RLD, and it is recommended to start them with an equivalent PSL dose ranging from 20 to 60 mg/day depending on the severity of the presentation [5–7].

We started treatment with intravenous PSL at 60 mg/day, and after 1 month, the lesion had shrunk, and symptoms, such as fever and cough, had improved. However, when the dose was reduced to 30 mg/day orally in the third month of PSL therapy, multiple nodular shadows appeared in both lungs on chest CT.

Previous reports have shown that malignancies tend to be associated with IgG4-RD. Wallace et al. reported that 20 of 125 patients diagnosed with IgG4-RD had a history of malignancy, with a standardized prevalence ratio as high as 2.5 [8]. Yamamoto et al. reported that 10.4% of patients with IgG4-RD had malignant complications, and the standardized incidence ratio was as high as 383 [9].

Based on the imaging findings of our case, the possibility of multiple lung metastases due to malignant disease was also suspected. To determine the treatment plan, thoracoscopic partial lung resection was performed on the newly appearing multiple nodules in the left lung. Extensive adhesions were also found in the left thoracic cavity, but they were more detachable than the adhesions in the right thoracic cavity. Early surgical intervention after the appearance of the lesions and the fact that the patient was taking PSL medication may have contributed to the mild adhesions. We found that IgG4-RLD refractory to PSL monotherapy showed shade changes from a solitary large mass (pseudotumor) to multiple nodules on chest CT. In addition, since the mass in the middle lobe was shrinking and the new shadow was not a malignant metastasis, the possibility of a malignant tumor was almost completely ruled out.

It has been reported that the combination of immunosuppressants and steroids is effective in treating IgG4-RLD refractory to PSL monotherapy [10], and we are considering the combination of azathioprine and PSL as a future treatment.

## Conclusion

We encountered a patient with IgG4-RLD refractory to PSL monotherapy with a change in shade from a solitary large mass (pseudotumor) to multiple nodules on chest CT. It was difficult to distinguish malignancy from IgG4-RLD, and the active surgical approach was useful in making the diagnosis and determining the treatment strategy.

## Data Availability

Not applicable.

## Abbreviations

IgG4: Immunoglobulin G4
IgG4-RD: IgG4-related disease
IgG4-RLD: IgG4-related lung disease
CT: Computed tomography
FDG-PET: ^18^F-fluorodeoxyglucose-positron emission tomography
SUVmax: Maximum standardized uptake value
PSL: Prednisolone
IL: Interleukin
HE: Hematoxylin–eosin
HPF: High power field
VATS: Video-assisted thoracic surgery
IHC: Immunohistochemistry
